# Evaluation of physicochemical and antioxidant properties of sourwood and other Malaysian honeys: a comparison with manuka honey

**DOI:** 10.1186/1752-153X-7-138

**Published:** 2013-08-12

**Authors:** Mohammed Moniruzzaman, Siti Amrah Sulaiman, Md Ibrahim Khalil, Siew Hua Gan

**Affiliations:** 1 Department of Pharmacology, School of Medical Sciences, Universiti Sains Malaysia, 16150 Kubang Kerian, Kelantan, Malaysia; 2 Human Genome Centre, School of Medical Sciences, Universiti Sains Malaysia, 16150 Kubang Kerian, Kelantan, Malaysia

**Keywords:** Sourwood, Longan, Gelam, Rubber tree honey, Antioxidants

## Abstract

**Background:**

The aim of the present study was to evaluate the physical, biochemical and antioxidant properties of four Malaysian monofloral types of honey (gelam, longan, rubber tree and sourwood honeys) compared to manuka honey.

Several physical parameters of honey, such as pH, moisture content, electrical conductivity (EC), total dissolved solids (TDS), color intensity, total sugar and sucrose content, were measured. A number of biochemical and antioxidant tests were performed to determine the antioxidant properties of the honey samples. Hydroxymethylfurfural (HMF) levels were determined using high performance liquid chromatography.

**Results:**

The mean pH, moisture content, EC and TDS of Malaysian honey were 3.90 ± 0.12, 17.01 ± 3.07%, 0.59 ± 0.17 mS/cm and 294.87 ± 81.96 ppm, respectively. The mean color and HMF level was 102.07 ± 41.77 mm Pfund and 49.51 ± 0.12 mg/kg, respectively. Sourwood honey contained the highest contents of phenolics (580.03 ± 0.38 mg_galic acid_/kg) and flavonoids (156.82 ± 0.47 mg_catechin_/kg) with high DPPH radical scavenging activity (59.26 ± 3.77%) as well as ferric reducing power [648.25 ± 0.90 μM Fe (II)/100 g]. Sourwood honey also exhibited the highest color intensity. Several strong positive correlations were observed amongst the different antioxidant parameters and the various antioxidant tests.

**Conclusion:**

This is the first time that the antioxidant potential of both sourwood and rubber tree honeys have been reported. Our results indicated that Malaysian honey (specifically sourwood honey and longan honey) is a good source of antioxidants compared to Manuka honey.

## Background

Honey is a natural product produced by honeybees and consists of a very concentrated solution of a complex mixture of sugars, in which fructose and glucose are the main ingredients [1]. Honey is a functional food and has different biological properties such as antibacterial (bacteriostatic properties), anti-inflammatory, wound and sunburn healing, antioxidant, radical scavenging, antidiabetic and antimicrobial activities [1-4].

In recent years, there has been an increasing interest in determining the antioxidant potentials of honey [5]. It has been reported that the botanical origin of honey has the greatest influence on its antioxidant activity, whereas processing, handling and storage can affect the antioxidant activity of honey only to a minor extent [2].

It has been shown in several studies that the antioxidant potential of honey is strongly correlated with the concentration of total phenolics present [1,2,5,6]. Furthermore, it has been reported that the antioxidant activity is also strongly correlated with the color of the honey, where dark colored honey has been reported to have a higher total phenolic content and consequently higher antioxidant capacities [2,5].

There are more than 150 polyphenolic compounds that have been reported, including phenolic acids, flavonoids, flavonols, catechins and cinnamic acid derivatives [7]. The composition and quantity of these components vary widely according to the floral and geographic origin of the honey. Several studies on the identification and quantification of the antioxidant components of honeybee products have been reported all over the world [7,8]. However, there is limited data available for Malaysian honey despite its high consumption rate by the general public.

Several types of honey are found in Malaysia. These are either directly or indirectly introduced in many different foods in Malaysia and have been used as a traditional medicine for the last few decades. Among the different types of honey available in the country, the antioxidant potentials of tualang and gelam honey have been previously reported [1,9-11].

Gelam honey is a wild monofloral honey produced by the *Apis dorsata* bees. The main nectar as well as the pollen collected by the bees are from the plant named *Melaleuca cajuputi Powell* or locally known as the “Gelam tree”. Gelam honey is produced in large amounts in the state of Terengganu on the eastern coast of peninsular Malaysia, where these mangrove trees grow abundantly. Longan honey is a monofloral honey produced by honeybees that acquire the nectar from the longan tree (*Dimocarpus longan* Lour.) flowers. Longan honey has an intense fragrant smell and is also known as “spring honey” because it is primarily produced in the springtime in other countries. Rubber tree honey is a monofloral honey produced by the *Apis mellifera* bees. The rubber tree (*Hevea brasiliensis*) is an abundant source of honey that is obtained from the extra-floral nectaries at the tip of the petiole where the leaflets join and has the rubber tree flavor. Contrary to its name, sourwood honey is not sour, but is sweet like any other honey. When consumed, this light-colored, delicate, subtle honey has an almost caramel or buttery flavor and a pleasant, lingering aftertaste. It is a monofloral honey produced from sourwood tree (*Oxydendrum arboreum*). The sourwood tree (also known as the sorrel tree) is the sole species in the genus *Oxydendrum*, in the family of *Ericaceae* (Table 1). Manuka honey from the manuka tree (*Lepto-spermum scoparium),* a native of New Zealand has been used as a standard for comparison in the present study. It is well known for various medicinal properties and its antioxidants, antibacterial, antifungal has been well established [12].

**Table 1:** Floral type and source of the investigated Malaysian honeys

**Name**	**Floral type and name of the Bees**	**Local and scientific name of the trees**
Gelam honey	Monofloral (*Apis dorsata*)	Gelam tree (*Melaleuca cajuputi*)
Longan honey	Monofloral (*Apis mellifera*)	Longan tree (*Dimocarpus longan*)
Rubber tree honey	Monofloral (*Apis mellifera*)	Rubber tree (*Hevea brasiliensis*)
Sourwood honey	Monofloral (*Apis mellifera*)	Sourwood tree or Appalachian Lily tree *(Oxydendrum arboretum)*

The aim of the present study was to investigate the physical parameters, chemical composition and the antioxidant potential of different types of Malaysian honey (gelam, longan, rubber tree and sourwood) and to compare these characteristics with Manuka honey. This research will help to identify the types of honey with high antioxidant activity that would promote beekeeping in Malaysia by increasing the commercial value of these honeys as a functional food source as well as a food additive. To our knowledge, this is the first time that data for sourwood, rubber tree and Malaysian longan honey have been reported.

### Experimental

#### Honey samples

Four different types of Malaysian honey samples (gelam, longan, rubber tree and sourwood) were selected. Each of the honey samples were acquired at 500 g for the present investigation. Gelam honey was supplied by the Federal Agriculture Marketing Authority (FAMA), Malaysia. Longan, rubber tree and sour wood honeys were supplied by the beekeepers from Perak, Malaysia. All honey collection was conducted between the months of July and September 2010. Manuka honey was used as a standard for comparison because it has been extensively studied. In this investigation, manuka Honey Active 5+ (Comvita®, New Zealand) was used. All the honey samples were stored at 4-5°C in airtight plastic containers until further analysis.

#### Chemicals and reagents

Ascorbic acid, bovine serum albumin (BSA), catechin, 2,2-diphenyl-1-picrylhydrazyl (DPPH), 2,4,6-tris(1-pyridyl)-1,3,5-triazine (TPTZ), 5-hydroxymethylfurfural (HMF), Folin–Ciocalteu’s reagent, gallic acid and proline were purchased from Sigma-Aldrich (St. Louis, Mo., U.S.A.). Sodium carbonate (Na_2_CO_3_), aluminum chloride (AlCl_3_), sodium nitrite (NaNO_2_) and sodium hydroxide (NaOH) were purchased from Merck (Darmstadt, Germany). All chemicals used were of analytical grade.

#### Physical analysis

##### pH

A pH meter (HI 98127, Hanna instruments, Mauritius) was used to measure the pH of a 10% (w/v) solution of honey prepared in Milli-Q water (Millipore Corporation, Billerica, Massachusetts, U.S.A.).

#### Moisture content

The moisture content was determined using a refractometric method. The refractive indices of the honey samples were measured at ambient temperature using an Atago handheld refractometer (KRUSS, HRH30, Hamburg, Germany) and the measurements were further corrected for the standard temperature of 20°C by adding a correction factor of 0.00023/°C. The percentage of moisture content corresponding to the corrected refractive index was calculated using a Wedmore’s table [13].

#### Total sugar content

Honey was suspended in Milli-Q water to produce a 25% (w/v) solution. The total sugar content of each honey sample was determined using the refractometric method (Atago handheld refractometer, ATAGO, N-1α, Tokyo, Japan) and the percentage of sucrose content was measured in g/mL of honey.

#### Electrical conductivity (EC) and total dissolved solids (TDS)

The EC and TDS were measured using a conductivity meter HI 98311 (Hanna Instruments, Mauritius) in a 20% (w/v) solution of honey suspended in Milli-Q water as recommended by [14]. The EC and TDS of each sample were analyzed and the means are expressed as mS/cm and ppm, respectively. The EC of milli-Q water alone was less than 10 μS/cm.

#### Honey color analysis

The color intensity of honey samples was measured according to the Pfund classifier. Briefly, homogeneous honey samples devoid of air bubbles were transferred into a cuvette with a 10-mm light path until the cuvette was approximately half full. The cuvette was inserted into a color photometer (HI 96785, Hanna Instruments, Cluj County, Romania) and the color grades were expressed in millimeter (mm) Pfund grades compared to an analytical grade glycerol standard. Measurements were performed for each sample using approved color standards of the United States Department of Agriculture (USDA) [15].

#### Color intensity (ABS_450_)

The mean absorbance of honey samples was determined using the method of [2]. Briefly, honey samples were diluted to 50% (w/v) with warm (45 -50°C) milli-Q water and the resulting solution was filtered using a 0.45 μm filter to remove large particles. The absorbance was measured at 450 and 720 nm using a spectrophotometer and the difference in the absorbance readings is expressed as mAU.

#### Determination of HMF levels by high-performance liquid chromatography (HPLC) method

HMF concentrations were determined using an HPLC method based on the method published by the International Honey Commission (IHC) [16]. Briefly, honey samples (10 g each) were diluted to 50 mL with distilled water, filtered using a 0.45 μm nylon membrane filter and injected (20 μL) into an HPLC system (Waters 2695, Milford, MA, U.S.A.) equipped with a Photodiode Array Detector (PDA) (Waters 2996). The HPLC column used was a Merck Purospher Star RP-18e (125 × 4 mm, 5 μm) fitted with a guard cartridge packed with similar stationary phase (Merck, Germany). The HPLC method included an isocratic mobile phase of 90% water and 10% methanol with a flow rate of 1.0 mL/min. All solvents used were of HPLC grade. The detection wavelength was 200–450 nm, with specific monitoring at 285 nm. The HMF concentrations in the samples were calculated by comparing the corresponding peak areas of the sample to the HMF standard solutions after correcting for the dilution of the honey samples. A linear relationship (r^2^ = 0.9997) was determined between the concentration and area of HMF peaks and the results are expressed in mg/kg.

#### Analysis of antioxidant potentials

##### Determination of total Phenolic content

The concentration of phenolics in the honey samples was estimated using a modified spectrophotometric Folin–Ciocalteu method [17]. Briefly, 2 g of honey was mixed with distilled water up to 10 mL. About 1 mL (0.2 g/mL) of honey extract was mixed with 1 mL of Folin and Ciocalteu’s phenol reagent. After 3 min, 1 mL of 10% Na_2_CO_3_ solution was added to the mixture and adjusted to 10 mL with distilled water. The reaction was kept in the dark for 90 min, after which the absorbance was read at 725 nm using a T 60 UV/VIS spectrophotometer (PG Instruments Ltd, UK). Gallic acid was used to calculate a standard curve (20, 40, 60, 80 and 100 μg/mL; r^2^ = 0.9970). The results are reported as the mean ± standard deviation and expressed as mg of gallic acid equivalents (GAEs) per kg of honey.

#### Determination of total flavonoid content

The total flavonoid content in each honey sample was measured using the colorimetric assay developed by [18]. Briefly, 2 g of honey was mixed with distilled water up to 10 mL. Honey extract (1 mL) was mixed with 4 mL of distilled water. At the baseline, 0.3 mL of NaNO_2_ (5% w/v) was added. After 5 min, 0.3 mL of AlCl_3_ (10% w/v) was added followed by the addition of 2 mL of NaOH (1 M) six min later. The volume was increased to 10 mL by adding 2.4 mL distilled water. The mixture was vigorously shaken to ensure adequate mixing and the absorbance was read at 510 nm. A calibration curve was created using a standard solution of catechin (20, 40, 60, 80 and 100 μg/mL; r^2^ = 0.9880). The results are expressed as mg catechin equivalents (CEQ) per kg of honey.

#### DPPH free radical-scavenging activity

The antioxidant potentials of each honey sample were studied by evaluating the free radical-scavenging activity of the DPPH radical, which is based on the method proposed by [7]. Briefly, 2 g of honey was mixed with distilled water up to 10 mL. About 0.5 mL (0.2 g/mL) of honey extract was mixed with 2.7 mL of methanolic solution containing DPPH radicals (0.024 mg/mL). The mixture was vigorously shaken and incubated for 15 min in the dark until the absorbance remained unchanged. The reduction of the DPPH radical was determined by measuring the absorbance of the mixture at 517 nm [19].

Butylated hydroxytoluene (BHT) was used as a reference. The radical-scavenging activity (RSA) was calculated as the percentage of DPPH discoloration using the following equation: *%* RSA = ([A_DPPH_–A_S_]/A_DPPH_) × 100, where A_S_ is the absorbance of the solution when the sample extract has been added at a particular concentration and A_DPPH_ is the absorbance of the DPPH solution.

#### Ferric ion reducing antioxidant power assay (FRAP assay)

The FRAP assay was performed according to a modified method described by [20]. Briefly, 200 μL of diluted honey (0.1 g/mL) was mixed with 1.5 mL of FRAP reagent. During the sample preparation, 1 g of honey was diluted with distilled water and was made up to 10 mL. The reaction mixture was then incubated at 37°C for 4 min and its absorbance was read at 593 nm against a blank that was prepared with distilled water. Fresh FRAP reagent was prepared by mixing 10 volumes of 300 mM/L acetate buffer (pH 3.6) with 1 volume of 10 mmol TPTZ solution in 40 mM/L HCl containing 1 volume of 20 mM ferric chloride (FeCl_3._6H_2_O). The resulting mixture was then pre-warmed at 37°C. A calibration curve was prepared using an aqueous solution of ferrous sulfate (FeSO_4_.7H_2_O) at 100, 200, 400, 600 and 1000 μM/L. The FRAP values were expressed as micromoles of ferrous equivalent (μM Fe [II]) per kg of honey.

#### Determination of ascorbic acid content

The ascorbic acid content was measured using the method described by [7]. A sample of the honey (100 mg) was extracted with 10 mL of 1% metaphosphoric acid at room temperature for 45 min and filtered through Whatman No. 4 filter paper. The filtrate (1 mL) was mixed with 9 mL of 0.005% 2,6-dichlorophenolindophenol (DCPIP) and the absorbance of the mixture was measured within 30 min at 515 nm against a blank. The ascorbic acid content was calculated based on a calibration curve of pure L-ascorbic acid (50, 100, 200 and 400 μg/mL; Y = 3.2453X - 0.0703; r^*2*^ = 0.9440). The results are expressed as mg of ascorbic acid/kg of honey.

#### Antioxidant content

The antioxidant content was determined by measuring AEAC (antioxidant equivalent ascorbic acid content) values using the method described by [6]. Honey samples were dissolved in methanol to a final concentration of 0.03 g/mL. A 0.75-mL aliquot of the methanolic honey solution was then mixed with 1.50 mL of a 0.02 mg/mL DPPH solution prepared in methanol. The mixture was incubated at room temperature for 15 min and the absorbance was measured at 517 nm using a spectrophotometer. The blank was composed of 0.75 mL of the methanolic honey solution mixed with 1.5 mL of methanol. Ascorbic acid standard solutions (1, 2, 4, 6 and 8 μg/mL) prepared in Milli-Q water were used to calculate the calibration curve (r^2^ = 0.978). The mean value is expressed as mg of ascorbic acid equivalent antioxidant content per 100 g of honey.

#### Proline content

The proline content in the honey samples was measured using a method established by the IHC [15]. Briefly, approximately 5 g of honey was transferred into a beaker and was dissolved in 50 ml water. The solution was quantitatively transferred to a 100 mL volumetric flask before further dilution to 100 mL with distilled water. After that, approximately 0.5 mL of the sample solution was transferred into a tube while 0.5 mL of water (blank test) was transferred into a second tube and 0.5 mL of proline standard solution were taken into three other tubes. To each tube, about 1 mL of formic acid and 1 mL of ninhydrin solution was added each. The tubes were capped carefully and shaken vigorously for 15 min. The tubes were then placed in a boiling water bath for 15 min and were immersed below the level of the solution. The tubes were further transferred to another water bath and incubated at 70°C for 10 min. About 5 mL of the 2- propanol water solution was added to each tube followed by immediate capping. The tubes were left to cool for about 45 min after its removal from the 70°C water bath and the absorbance were measured at 510 nm (near maximum).

#### Biochemical analyses

##### Protein content

The protein content of honey was measured according to Lowry’s method [21]. Briefly, BSA solutions were prepared by diluting a stock BSA solution (1 mg/mL) to 5 mL. BSA concentrations ranged from 0.05 to 1.00 mg/mL. Based on these dilutions, 0.2 mL of protein solution was placed in different test tubes and 2 mL of alkaline copper sulfate reagent (analytical reagent) was added. After the resulting solution was mixed properly, it was incubated at room temperature for 10 min. Then, 0.2 mL of Folin–Ciocalteu reagent solution was added to each tube and incubated for 30 min. The colorimeter was calibrated with a blank and the absorbance was measured at 660 nm.

#### Reducing sugar assay

The total reducing sugar content was measured using 3,5-dinitrosalicylic acid (DNSA). The reducing sugar reduces DNSA to 3-amino-5-nitrosalicylic acid, resulting in a solution with reddish-orange coloration that is measured spectrophotometrically at 540 nm [22]. The honey solution (0.1 g/mL) was diluted 100-fold with Milli-Q water. A 1-mL aliquot of this diluted solution was mixed with equal amounts of DNSA solution and incubated in a boiling water bath for 10 min. The mixture was allowed to cool to ambient temperature for 10 min and mixed with 7.5 mL of Milli-Q water followed by measurement of the absorbance at 540 nm using a spectrophotometer. Glucose solutions of known concentrations (100, 200, 400 and 600 μg/mL) were used as standards.

The amount of non-reducing sugars, such as sucrose content (%), was measured by subtracting the reducing sugar content from total sugar content, which is expressed by the following equation:

$$\mathrm{Sucrose}\;\mathrm{content}\;\left(\%\right)=\mathrm{Total}\;\mathrm{sugar}\;\mathrm{content}-\mathrm{Reducing}\;\mathrm{sugar}$$

#### Statistical analysis

The assays were performed in triplicate and the results are expressed as the mean values with standard deviations (SD). The significant differences represented by letters were obtained by a one-way analysis of variance (ANOVA) followed by Tukey’s honestly significant difference (HSD) post hoc test (p < 0.05). Correlations were established using Pearson’s correlation coefficient (r) in bivariate linear correlations (p < 0.01). These correlations were calculated using Microsoft office Excel 2007 and SPSS version 16.0 (IBM corporation, New York, U.S.A.).

## Results and discussion

### Analysis of the physical properties of honey

#### pH and moisture content of honey

All of the Malaysian honeys analyzed in this study were found to be acidic (Table 2). The mean pH values determined for Malaysian honeys were 3.85 ± 0.04, whereas manuka honey was less acidic at pH 4.10. The pH values of the Malaysian honey samples were similar to those reported for Algerian, Brazilian, Bangladeshi, Indian and Spanish honeys (between pH 3.49 and 4.70) [4,22-25]. It has been reported that the high acidity of honey is caused by the fermentation of sugar into organic acid, which has been reported to be responsible for honey’s flavor and stability against microbial spoilage [26]. Overall, the pH values of the studied honey samples were within the limit that indicated the freshness of the honey samples (between pH 3.4 and 6.1) as described in the literature [25].

**Table 2:** Physical parameters (pH, moisture, sucrose, electrical conductivity, total dissolved solids content and color characteristics) of various Malaysian honeys

**Sample**	**pH**	**Moisture content**	**EC**	**TDS**	**ABS**_**450**_
		**(%)**	**Mean ± SD**	**Mean ± SD**	**(mAU,50 w/v)**
		**Mean ± SD**	**mS/cm**	**ppm**	**Mean ± SD**
Gelam	3.83 ± 0.06^b^	17.93 ± 0.23^b^	0.74 ± 0.011^b^	368.33 ± 5.86^b^	585.33 ± 5.51^d^
Longan	3.83 ± 0.06^b^	18.59 ± 0.12^a^	0.48 ± 0.005^d^	242.33 ± 3.21^d^	660.67 ± 3.06^c^
Rubber Tree	3.83 ± 0.06^b^	19.06 ± 0.20^a^	0.41 ± 0.001^e^	206.67 ± 0.58^e^	204.67 ± 5.03^e^
Sourwood	3.90 ± 0.00^b^	17.86 ± 0.40^b^	0.79 ± 0.009^a^	394.33 ± 4.62^a^	713.67 ± 5.51^b^
Manuka	4.10 ± 0.00^a^	11.59 ± 0.12^c^	0.53 ± 0.002^c^	262.67 ± 0.58^c^	805.00 ± 13.45^a^
Mean ± SD	3.90 ± 0.12	17.01 ± 3.07	0.59 ± 0.17	294.87 ± 81.96	593.87 ± 231.81

Means are compared by using One way ANOVA-Post Hoc Multiple Comparisons. In each column, values with different letters (superscripts “a-e”) indicate significant differences (*p <* 0.05).

The moisture content (%) in the investigated samples ranged from 11.59 to 19.06. The moisture content of rubber tree honey was slightly higher (19.06%), which may be due to its different floral source. All of the tested Malaysian honeys had moisture contents below 20%, which is the maximum prescribed limit (≤20%) for the moisture content as per the international regulations for honey [27,28]. The moisture content in manuka honey was 11.59% which was the lowest among all types of honey tested (Table 2) which allows manuka honey to be protected from microbial attacks during long time of storage [29].

Moisture content is a vital factor for honey quality because a higher moisture content could lead to undesirable fermentation of the honey during storage caused by the action of osmotolerant yeasts, which results in the formation of ethyl alcohol and carbon dioxide. The alcohol can be further oxidized to acetic acid and water, which leads to a sour taste [30]. Moreover, the moisture content of honey depends on various factors such as the harvesting season, the degree of honey maturity in the hive and climatic factors [31]. Low moisture content has been reported to be advantageous, as it can promote a longer shelf life during storage [32].

The moisture content of the analyzed samples was consistent with the previously reported values of some Malaysian honeys for which the corresponding values ranged from 12.79% to 22.32% [33] and 14.86% to 17.53% [29]. Furthermore, the moisture contents for Malaysian honeys were similar to those of other honeys, including Portuguese honey (15.9-17.2%) [3], Moroccan honey (14.3 to 20.2%) [34] and Indian honey (17.2-21.6%) [22]. Overall, the low moisture content in our investigated honey samples suggests that they are of good quality and storage capability.

#### Electrical conductivity (EC) and total dissolved solids (TDS)

EC is a key physicochemical parameter for the authentication of unifloral honeys [24]. The EC value depends on the ash and acid content in honey in which the higher the content, the higher the resulting conductivity [16]. This parameter was recently included in the international standards, replacing the determination of ash content [27].

The EC values in the investigated honey samples varied in the range of 0.41-0.79 mS/cm and were within the recommended range (lower than 0.8 mS/cm) (Table 2). The EC value is similar to the EC values for manuka honey (0.53 mS/cm). The EC values of some Algerian honeys were reported to be higher (0.21-1.61 mS/cm) in a previous study by [4]. However, our results are similar to the EC values previously reported in India [22], Bangladesh [23] and Morocco [32]. These variations could be due to differences in the geographical origin of the honey.

TDS is a measure of the combined content of all inorganic and organic substances in honey such as molecular, ionized or micro-granular (colloidal solution) suspended forms. The TDS values of Malaysian honeys ranged between 206.67 and 368.33; comparable to that of manuka honey (262.67 ppm). Our results demonstrated that there is a correlation between EC and TDS, suggesting that both parameters are good indicators for honey purity. Sourwood honey showed the highest EC (0.79 mS/cm) and the highest content of TDS (394.33 ppm), which indicates that it is rich in both organic and inorganic substances.

#### Total sugar content

Overall, the total sugar content of Malaysian honey samples in the present study was between 55.33 and 64.93% (Table 3). In our study, the total sugar content of sourwood and longan honey was lower (at 55.33 and 56.67%, respectively); these results are similar to those reported for Indian honey, which ranged from 43.3 to 66.7% [22] and Bangladeshi honey, which ranged from 42.80% to 60.67% [23]. The lower total sugar content can be contributed by the conversion of sugar into inorganic acid. It has also been reported that overheating of honey samples during processing or storage for very long periods can lead to the conversion of sugars to HMF [22]. The other two Malaysian honeys (gelam and rubber tree honeys) had higher total sugar content at 64.93% and 62.27%, respectively, whereas manuka honey contained 60.93%. To our knowledge, Algerian honey has been reported to have the highest total sugar content at 62.80 to 70.00% [25] and 71.25 to 84.25% [4].

**Table 3:** Reducing and non-reducing sugar content of Malaysian honeys

**Sample**	**Total sugar content % (g/100 g) mean ± SD**	**Reducing sugar (%) g/100 g mean ± SD**	**Sucrose (%) g/100 g mean ± SD**
Gelam	64.93 ± 1.22^a^	62.17 ± 0.73^a^	2.77 ± 1.47^a^
Longan	56.67 ± 1.22^c^	54.78 ± 0.51^d^	1.89 ± 0.71^a^
Rubber Tree	62.27 ± 0.46^b^	60.61 ± 0.25^b^	1.66 ± 0.71^a^
Sourwood	55.33 ± 1.22^c^	52.17 ± 0.44^e^	3.17 ± 0.79^a^
Manuka	60.93 ± 0.46^b^	58.61 ± 0.42^c^	2.32 ± 0.66^a^
**Mean ± SD**	60.03 ± 3.98	57.67 ± 4.14	2.36 ± 0.62

Means were compared using a one way ANOVA with post hoc multiple comparisons. In each column, values with different letters (superscripts “a-e”) indicate significant differences (*p <* 0.05).

#### Color characteristics

Color is the primary characteristic for honey classification and is classified according to USDA-approved color standards [15]. The color of honey varies naturally, ranging from light yellow to amber, dark amber and black in extreme cases and sometimes even green or red hues [35]. Honey usually darkens with age. Other changes in color may result from the beekeeper’s interventions and different ways of handling the combs such as the use of old honeycombs, contact with metals and exposure to either high temperatures or light. Because the color of untreated honey depends on its botanical origins, color classification of monofloral honeys is very important for commercial activities.

**Figure 1:**
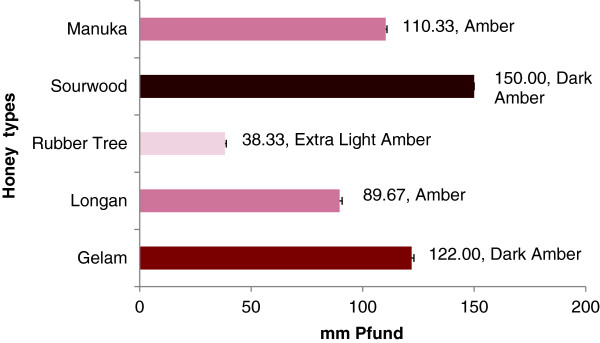
Color characteristics of different Malaysian honeys.

In the present study, sourwood honey had the highest Pfund value (150 mm Pfund) and had a dark amber color. The Pfund value of sourwood honey is similar to Algerian honey [25] and some Bangladeshi honeys [23], which have been reported to have more antioxidant properties, which suggests that sourwood honey may also have a significant amount of antioxidant properties. Gelam and manuka honeys were amber, with Pfund values of 122 and 110, respectively (Figure 1).

#### Color intensity

It has been reported that differences in honey origin and composition are significantly reflected in their color intensities [36]. Therefore, honey color is an important parameter and can be used in the identification of its floral origin. The color intensities (ABS_450_) of the analyzed honeys ranged between 204 and 805 mAU (Table 2). Manuka honey had the highest color intensity (805 mAU) indicating its high antioxidant potential. Among the Malaysian honey samples, sourwood honey had the highest level (713.67), which suggests that it has the highest antioxidant potential among the Malaysian honey.

When compared with the honey samples from other countries, the ABS_450_ values were reported to be between 25 and 3413 mAU in Italian honey [2], 724 and 1188 mAU in Algerian honey [25], 70 and 495 mAU in Slovenian honey [5], 254 and 2034 mAU in Bangladeshi honey [23] and 524 and 1678 mAU in Indian honeys [22]. ABS_450_ is a reliable parameter for confirming the presence of pigments that have antioxidant activities such as carotenoids and some flavonoids and ABS_450_ is usually correlated with the phenolic levels and flavonoid content of the honey. This is also true for our study in which honey samples with higher phenolic and flavonoid content tend to have significantly higher color intensities, as observed with sourwood honey.

#### HMF content of honey

HMF is an important indicator for honey purity, as HMF content is widely recognized as a parameter that indicates the freshness of honey [36]. High concentrations of HMF in honey are an indicator of overheating and storage in poor conditions. According to the Codex Alimentarius Commission [26], the HMF concentration in honey should not exceed 80 mg/kg [27]. Aside from storage conditions (e.g., temperature), the age of the honey and floural sources can also influence HMF levels [36-38]. It has been reported that HMF concentrations of honey stored for longer periods (12–24 months) increased to significant amounts (Khalil et al., 2010) that exceed the recommended levels that are considered to be suitable and safe for human consumption.

**Figure 2:**
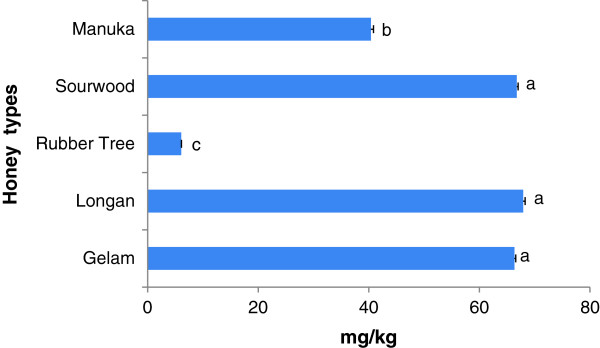
**HMF concentrations found in different Malaysian honeys.** Results are expressed as Mean + SD. The different letters indicate statistically significant differences (*p <* 0.05).

The HMF concentrations in the investigated Malaysian honey samples ranged from 6.07 to 67.94 mg/kg (Figure 2) and are within the limit set by the Codex Alimentarius Commission and the European Union. The slightly higher HMF concentration in gelam, longan and sourwood honey may be due to conversion of sugars (sucrose) into HMF because it has been previously reported that HMF can also be produced from decomposition of hexoses catalyzed by heating [39] and these honey samples coincidentally had high sucrose concentrations. Another factor that can affect the HMF content of honey is the tropical climate of Malaysia because it has been reported that hot weather can increase the HMF levels of honey in the bee hives themselves [40]. This is the reason why the European Union [41], which recommended a lower limit of 40 mg/kg, allowed a higher limit of 80 mg/kg for honey that originates from countries or regions with tropical temperatures, including Malaysia.

HMF levels of the investigated honey samples were much lower than the previously reported HMF content of Malaysian tualang and gelam honey (between 6.65 to 1131.76 mg/kg) [33], which is indicative of their high quality. Our result is similar to the reported HMF levels of honeys originating from other tropical countries such as Morocco (0.09 to 53.38 mg/kg) [34] and Australia (50.8 to 74.9 mg/kg) [40]. As expected, the HMF concentration is higher than in honey samples originating from cooler countries such as Portugal (1.75 to 32.75 mg/kg) [38].

#### Antioxidant analyses

##### Total Phenolic content

**Figure 3:**
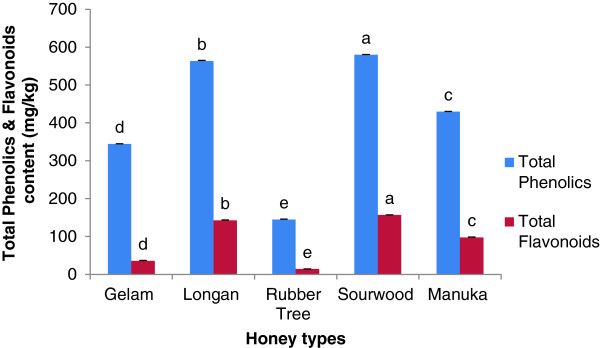
**Total phenolic and flavonoids content of Malaysian honeys.** Results are expressed as Mean + SD. The different letters indicate statistically significant differences (*p <* 0.05).

Polyphenols, represented by the total phenolic content, are an important group of compounds that were reported to influence not only the appearance but also the functional properties of honey [42]. The total phenolic content of the honeys tested in the present study was between 144.51 and 580.03 mg_galic acid_/kg of honey (Figure 3) and a significant difference was observed in the phenolic contents of the different honey types. Sourwood honey contained the highest phenolic content (580.03 mg/kg) followed by longan honey (563.55 mg/kg), both of which were higher than the phenolic content in manuka honey (429.61 mg/kg) indicating that both sourwood and longan honeys have better antioxidant potentials.

The phenolic content of sourwood and longan honey is higher than that of Slovenian fir and forest honey at 241.4 and 233.9 mg/kg [5], some Algerian honey (411.10 to 498.16 mg/kg) [25], Indian rain forest honey (456.30 mg/kg) [10], Bangladeshi honey (152.4 to 688.5 mg/kg) [23], morning glory honey from Cuba (347.5 mg/kg), black mangrove honey (233.6 mg/kg) and Christmas vine honey (213.9 mg/kg) [43]. The high phenolic contents of both sourwood and longan honeys may be due to the higher amounts of these substances present in the sourwood and longan trees. The phenolic contents of different parts of Longan tree (*Dimocarpus longan* Lour) were also reported to be different when they were investigated [44].

In particular, sourwood honey contained a higher phenolic content compared to other previously reported Malaysian honeys such as tualang honey (251.7 ± 7.9 mg/kg) [11], pineapple honey (277.5 mg/kg) [10] and Manuka honey (52*.*63 ± 1*.*21 mg/100 g) [9], which suggests that sourwood honey has a high antioxidant potential.

The determination of the total phenolic content has also been regarded as a promising method of studying the floral origins of honeys [45]. It has been reported that the botanical and geographical region from which the honey is collected not only affects the phenolic and flavonoid concentrations but also pollen distribution and the eventual antioxidant activities of the honey. Many studies have also reported that the plant source can result in significant differences in floral honeys [2,5].

#### Total flavonoid content

Flavonoids are low molecular weight phenolic compounds responsible for the aroma and antioxidant potential of honey. The total flavonoid content in the tested honey samples ranged from 14.20 to 156.82 mg_catechin_/kg (Figure 3). As with the phenolic content, sourwood honey showed the highest levels of flavonoid content (156.82 mg/kg) among all of the types of honey investigated. The flavonoid concentration of longan honey (142.63 mg/kg) was second highest and was also higher than that of manuka honey (97.62 mg/kg) indicating its high antioxidant potential.

The flavonoid concentration of these honeys were higher than that of Algerian honey [25]; Indian forest honey [10]; fir, lavender, ivy and acacia honey from Cuba [46]; Bangladeshi honeys [23] and tualang, gelam and pineapple honey from Malaysia [9,10]. The higher flavonoid content present in sourwood and longan honeys suggests their superior antioxidant capabilities.

Flavonoids are the predominant phenolic class present in honeybee-collected pollen and are best described for their ability to act as antioxidants [47], with one of the best-known mechanisms by direct scavenging of free radicals. Flavonoids are oxidized by radicals, resulting in a more stable, less-reactive radical. Flavonoids stabilize reactive oxygen species by neutralizing with the reactive element of the radical [48]. Therefore, honey containing higher flavonoid concentrations is desirable due to their purported antioxidant potential.

#### DPPH radical scavenging assay

The radical scavenging activities of the honey samples were determined by using the DPPH radical scavenging assay. DPPH is a stable nitrogen-based radical that has been extensively used to test the free radical scavenging ability of various substances. In evaluating the radical-scavenging potential of honey, the DPPH assay is frequently used. Usually, a high DPPH scavenging activity confers the high levels of antioxidant activity of the sample.

**Figure 4:**
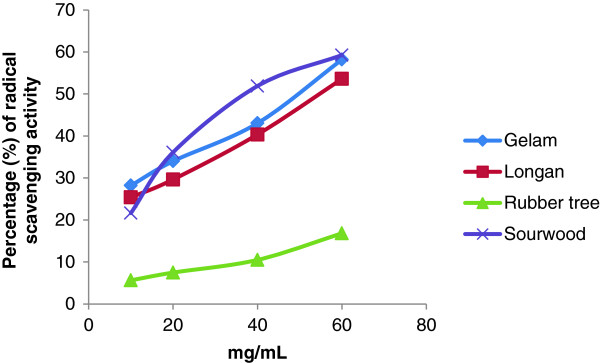
Percentage of inhibition of DPPH radical scavenging activity at different concentrations.

The DPPH radical scavenging activities of all of the honey samples were measured at the following concentrations: 10, 20, 40 and 60 mg/mL. The highest percentage of inhibition was observed at 60 mg/mL for all of the honey samples (Figure 4). Sourwood honey had the highest DPPH radical scavenging ability (59.26%), further supporting the possibility that it contains the highest amount of free-radical scavenging compounds and the highest antioxidant potential. Its high radical scavenging activity may be due to its high phenolic and flavonoid content because the antioxidant potential of honey has been reported to be directly proportional with amount of phenolics and flavonoids present [2]. Overall, both the DPPH scavenging and antioxidant potential of Malaysian honey is higher than that previously reported for some Malaysian gelam and Borneo tropical honeys [9], Indian honey [22] and Algerian honey [25].

#### FRAP assay

The FRAP assay is used to determine the total antioxidant content of honey. The assay directly estimates the presence of either antioxidants or reductants in a sample, depending on the ability of the analyte to reduce the Fe^3+^/Fe^2+^ couple [2]. The FRAP values for the tested Malaysian honey ranged from 209.78 to 653.75 μM Fe (II)/100 g of honey.

There were significant differences among FRAP values of the different types of honey (Table 4), suggesting that they have different antioxidant potentials. Again, sourwood honey had the highest FRAP values among all the investigated honey, which indicates its significant reducing power and antioxidant potential. The antioxidant activity for the different types of honey decreased as follows: sourwood > manuka > longan > gelam > rubber tree honey. Moreover, the FRAP value of sourwood honey is higher than that of Slovenian fir honey [478.5 μM Fe(II)] [5], forest honey [426.4 μM Fe(II)] [5], Cuban honey [196.7 μM Fe(II)] [46], Algerian honey [403.54 μM Fe(II)] [25] and Indian forest honey [73.35 μM Fe(II)] [10], as well as acacia [79.5 μM Fe(II)], chestnut [388.6 μM Fe(II)] and Chicory honeys [209.5 μM Fe(II)] [2]. The FRAP value of sourwood honey is also higher than that reported in some Malaysian tualang honey [322.1 μM Fe(II) [11] and 576.91 ± 0.64 μM Fe (II) [29]], pineapple honey [47.92 μM Fe (II)] and gelam honey [115.61 μM Fe (II)] [10], which suggests that sourwood honey has the highest level of antioxidant activity.

**Table 4:** Biochemical and antioxidant properties of Malaysian honeys

**Sample**	**FRAP values mean ± SD (μM Fe (II)/100 g)**	**Proline mean ± SD (mg/kg)**	**Protein mean ± SD (g/kg)**
Gelam	325.79 ± 1.55^d^	261.33 ± 1.33^c^	3.14 ± 0.01^c^
Longan	426.38 ± 0.49^c^	184.96 ± 0.64^d^	2.94 ± 0.02^d^
Rubber Tree	209.78 ± 1.20^e^	184.75 ± 0.98^d^	2.14 ± 0.02^e^
Sourwood	653.75 ± 0.71^a^	498.56 ± 0.64^b^	5.59 ± 0.01^a^
Manuka	648.25 ± 0.90^b^	564.91 ± 1.33^a^	5.04 ± 0.02^b^
**Mean ± SD**	452.79 ± 196.51	338.90 ± 0.35	3.77 ± 1.47

Means were compared by using one-way ANOVA with post hoc multiple comparisons. In each column, values with different letters (superscripts “a-e”) indicate significant differences (*p <* 0.05).

#### Ascorbic acid and AEAC contents

**Figure 5:**
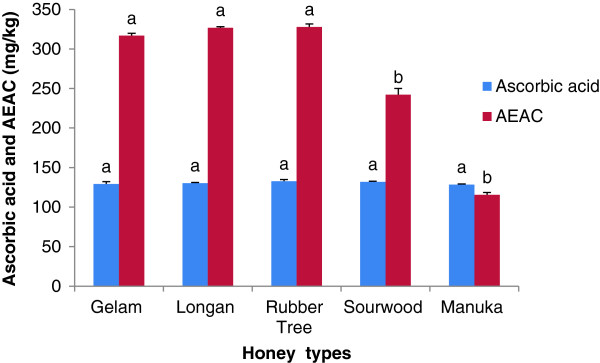
**Ascorbic acid and AEAC contents of different Malaysian honeys.** Results are expressed as Mean + SD. The different letters indicate statistically significant differences (*p <* 0.05).

Aside from polyphenols, ascorbic acid is one of the non-enzymatic substances present in honey that is a known antioxidant [46]. The ascorbic acid content of Malaysian honey ranged from 129.14 to 132.68 mg/kg (Figure 5). Among all of the investigated honey samples, rubber tree honey showed the highest ascorbic acid content at 132.68 mg/kg of honey. This high ascorbic acid content may be attributed to the presence of high vitamin C present in rubber tree (*Hevea brasiliensis).*

Sourwood honey had a slightly higher (132.07 mg/kg) ascorbic acid concentration compared to manuka honey (128.9 mg/kg) which corroborate its sour taste. The ascorbic acid concentration was also high in Bangladeshi honey (129.8 to 154.3 mg/kg) [23], Portuguese honey (140–145 mg/kg) [7], Indian forest honey (260.90 mg/kg) [10], Algerian honey (236.80 to 315.90 mg/kg) [25] and Malaysian pineapple honey (146.40 mg/kg) [10]. Generally, ascorbic acid concentrations decrease as the duration of storage increases. It has also been reported that when honey is stored for a long duration, the concentrations of several other compounds may also decrease, which can affect both ascorbic acid and enzyme levels [49]. These factors can lead to variations in the ascorbic acid concentrations of honey.

The AEAC content of the Malaysian honey samples was measured as mg of AEAC/kg of honey using an ascorbic acid standard curve and ranged from 242.23 to 327.97 mg of AEAC/kg; however, the content was lower (115.68 mg/kg) in manuka honey (Figure 5). These values are similar to those of honeys from Burkina Faso [6] and Algeria [25], but Indian honey samples had lower values (between 151 and 295 mg of AEAC/kg) [22].

#### Biochemical properties

##### Reducing sugar and sucrose contents

The major sugars present in honey are fructose and glucose. The reducing sugar content of all of the Malaysian honeys tested was between 52.17 and 62.17%. Sourwood and longan honey had a lower amount of reducing sugar compared to the limit (≥60 g/100 g) set by the European community directive [28]. One possible reason may be the conversion of sugars into organic acids. However, the levels of reducing sugars in gelam, longan and manuka honeys were within the limit. Our results indicated that reducing sugars are the primary soluble sugars present in Malaysian honey samples.

The sucrose (saccharose) contents of the tested Malaysian honey ranged from 1.66 to 3.17%. These values were within the maximum prescribed limit of sucrose content for honey (5%) recommended by the Codex standard [27]. The sucrose content of the Malaysian honey tested in this study is similar to those observed in Moroccan honey (0.42 to 2.98%) [34] and Algerian honey 0.08% to 5.31% [4]. The variations in the sucrose levels may be indicative of the effect that different regions have on the compositional differences of honey. In future studies, the sugar content of these honeys could be analysed by using chromatographic methods.

#### Proline content

Proline is produced primarily from the salivary secretions of *Apis mellifera* during the conversion of nectar into honey. It is the major component (50–80%) of the total amino acids present in honey [50]. The concentration of proline ranged between 184.75 and 564.91 mg/kg (Table 4). The proline content of honey should normally be more than 200 mg/kg [26]. The proline content of Malaysian honey is similar to that of Algerian (202 to 608 mg/kg) [4], Indian (133–674 mg/kg) [22] and Bangladeshi honey (106–681 mg/kg) [23]. Manuka honey contained the highest concentration of proline (564.91 mg/kg) followed by sourwood (498.56 mg/kg) and gelam honey (261.33 mg/kg). The high levels of proline in all of the studied honey samples are suggestive of their ripeness and discount the possibility of sugar modification or adulteration. This is because the proline content is a sign of honey ripeness and high proline content indicates a lower probability of honey adulteration [26].

#### Protein content

The total protein content of honey is dependent on the flower sources and can be subsidized by the enzymes introduced by either the bees or other substances derived from the nectar [43]. The total protein content of the investigated honey samples ranged from 2.14 to 5.59 g/kg (Table 4).

Sourwood honey had the highest concentration of protein compared to manuka honey and other Malaysian honey samples. The protein content of honey is normally less than 5.00 g/kg [22]; however, the protein content of sourwood honey (5.59 g/kg) was slightly higher than the recommended value. It is possible that the sourwood tree (*Oxydendrum arboretum*) produce large amounts of pollen and nectar, which can contribute to the protein content in the honey samples. However, this requires further investigation. A high protein concentration has also been reported in some Algerian honey (3.7 to 9.4 g/kg) [4]. The protein content in other Malaysian honey samples was similar to honey samples from India (2.29 g/kg) [22].

#### Correlations amongst biochemical parameters and antioxidant potentials

Several strong correlations were established amongst several biochemical and antioxidant parameters. A strong correlation was found between the color intensity of honey samples and the antioxidant parameters, phenolics, flavonoid, proline and protein contents at 0.837, 0.735, 0.701, 0.938 and 0.873, respectively, as well as with the DPPH and FRAP values (Table 5). The color intensity of honey also increased with increases in the phenolic and flavonoid contents of the honey. For example, sourwood honey, which had the highest color intensity, also exhibited the highest phenolic content. This finding suggests that honey color pigments may have a role in the observed antioxidant activities of honey samples.

**Table 5:** **Correlation matrix showing the interrelation among phenolics, flavonoids, DPPH scavenging, FRAP, ascorbic acid, proline content, ABS**_**450 **_**and protein levels**

	**Phenolics**	**Flavonoids**	**DPPH**	**FRAP**	**Ascorbic acid**	**Proline**	**ABS**_**450**_	**Protein**
Phenolics	1.000	0.958**	0.789*	0.761*	0.158	0.419	0.837**	0.647*
Flavonoids	0.958**	1.000	0.607*	0.782*	0.103	0.443	0.735*	0.659*
DPPH	0.789**	0.607*	1.000	0.671*	0.542*	0.479	0.938**	0.590*
FRAP	0.761*	0.782*	0.671*	1.000	0.216	0.900**	0.873**	0.960**
Ascorbic acid	0.158	0.103	0.542*	0.216	1.000	0.229	0.468	0.151
Proline	0.419	0.443	0.479	0.900**	0.229	1.000	0.701*	0.947**
ABS_450_	0.837**	0.735*	0.938**	0.873**	0.468	0.701*	1.000	0.783*
Protein	0.647*	0.659*	0.590*	0.960**	0.151	0.947**	0.783*	1.000

**Correlation is significant at the 0.01 level (2-tailed); *Correlation is significant at the 0.05 level (2-tailed).

Another strong correlation was established between ABS_450_, DPPH and FRAP values, suggesting the involvement of pigments that confer antioxidant potential to honey. In a previous study conducted by [5] a strong correlation (r = 0.850) between the ABS_450_ and FRAP values was established in Slovenian honeys. The correlation between the ABS_450_ and FRAP values was also high (r = 0.83) in Indian honeys [22], which indicates that ABS_450_, DPPH and FRAP values are good predictors for antioxidant properties of honey. Thus, the higher correlations calculated in our study (ABS_450_ & DPPH, r = 0.938; ABS_450_ & FRAP, 0.873) suggest that Malaysian honeys have a stronger antioxidant capacity compared to Indian and Slovenian honeys.

A positive significant linear correlation was also observed between the following antioxidant parameters: 1) phenolic and flavonoid content with DPPH radical scavenging activity and 2) phenolic and flavonoid content with FRAP values. Overall, the positive correlations between DPPH and total phenolic content suggest that phenolics are the strongest contributing factor to the radical scavenging activity of Malaysian honeys.

Proline, an important amino acid that confers antioxidant potential to honey, also strongly correlates with FRAP, ABS_450_ and protein content. The most significant correlation was observed between proline content and protein content values (r = 0.947), suggesting that the proline content also contributes to the antioxidant potential of Malaysian honey. The correlation between the protein content and the FRAP values was 0.960, indicating that the protein content of honey may have some role in the antioxidant potential of honey. Overall, these strong positive correlations clearly suggest that Malaysian honey samples have strong antioxidant potential.

## Conclusion

This is the first report on the physicochemical and antioxidant potentials of sourwood, Malaysian longan and rubber tree honeys. Our results clearly indicate that sourwood honey possesses the best antioxidant effects when compared with gelam, longan and rubber tree honeys as well as manuka honey. This study showed that the phenolic, flavonoid, ascorbic acid and proline contents of honey are responsible for its free radical scavenging and antioxidant activity. Furthermore, several strong positive correlations were observed amongst the different antioxidant markers and antioxidant test values, which demonstrated the overall antioxidant properties of Malaysian honeys. Sourwood honey, which contained the highest concentrations of phenolics, flavonoids and ascorbic acid, is the best source of antioxidants and should be more widely consumed.

## Abbreviations

(BSA): Bovine serum albumin; (DPPH): 2,2-Diphenyl-1-picrylhydrazyl; (TPTZ): 2,4,6-Tris(1-pyridyl)-1,3,5-triazine; (HMF): 5-Hydroxymethylfurfural; (FRAP): Ferric ion reducing antioxidant power assay; (AEAC): Antioxidant equivalent ascorbic acid content.

## Competing interests

There is no conflict of interest statement among the authors.

## Authors’ contributions

MM carried out the experimental parts of this investigation and prepared the manuscript. MIK helped to conduct the study. SAS, and SHG supervised the work, evaluated the results and corrected the manuscript for publication. All authors read and approved the final manuscript.
